# Exploring the Impact of Dietary Factors on Intestinal Diverticular Disease: A Mendelian Randomization Approach

**DOI:** 10.1002/fsn3.4623

**Published:** 2024-12-01

**Authors:** Wangzhao Tian, Wenwen Yang, Xiaorui Wang, Yanjiang Yang

**Affiliations:** ^1^ The Central Hospital of Enshi Tujia and Miao Autonomous Prefecture Enshi Hubei China; ^2^ The First Clinical Medical College, Lanzhou University Lanzhou Gansu China; ^3^ School of Pharmacy, Chengdu University Chengdu China; ^4^ Department of Rheumatology and Immunology The people's Hospital of Qiandongnan Autonomous Prefecture Kaili Guizhou China

**Keywords:** diet, dried fruit intake, intestinal diverticular disease, Mendelian randomization

## Abstract

Dietary habits significantly influence the development of intestinal diverticular disease (IDD), a common gastrointestinal condition primarily affecting the colon. We performed a Mendelian randomization (MR) analysis on 20 diet‐related factors using data from the UK Biobank. IDD cases (*n* = 33,618) and controls (*n* = 329,381) were obtained from the FinnGen Biobank. Three key MR methods were applied: the inverse‐variance‐weighted (IVW) method as the primary approach to estimate causal relationships, along with the weighted median (WM) and MR‐Egger methods. Significant associations were found for pork intake (*β* = 1.06, *p* = 0.00244), nonoily fish intake (*β* = 0.709, *p* = 0.0449), oily fish intake (*β* = 0.246, *p* = 0.0222), and dried fruit intake (*β* = −0.953, *p* < 0.0001). After false discovery rate (FDR) adjustment, pork intake (*q* = 0.0244) and dried fruit intake (*q* < 0.0001) remained significant. Our results indicate that while pork and certain types of fish intake may elevate the risk of IDD, dried fruit intake may offer a protective effect. These findings highlight the potential of dietary changes in IDD prevention and management, though further research across diverse populations is needed.

## Graphical abstract

The study employed Mendelian randomization to explore the impact of 20 dietary factors on intestinal diverticular disease (IDD). It found that pork and fish consumption (both oily and nonoily) were associated with an increased risk of IDD, while dried fruit intake was protective. After adjusting for confounding factors, dried fruit intake remained significantly linked to reduced IDD risk, highlighting the potential for dietary modifications in IDD prevention.

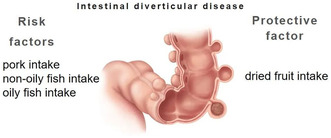

## 1 Introduction

Diverticula, small sac‐like protrusions that form through weak spots in the intestinal wall, are among the most common anatomical changes in the human intestine (Tursi et al. 2020), encompassing diverticular disease—a spectrum of conditions primarily affecting the colon, where these pouches can exist without inflammation (diverticulosis) or become inflamed and infected (diverticulitis). Diverticular disease is a highly prevalent gastrointestinal disorder that is widely distributed in Western countries (Cuomo et al. 2014; Peery et al. 2015; Etzioni et al. 2009; Barbaro et al. 2022). The lifestyle has long been regarded as a key factor in the development of diverticular disease (Tursi et al. 2020). Burkitt and Painter initially described diverticular disease as a condition that could potentially be prevented through dietary modifications (Burkitt, Walker, and Painter 1972). Currently, numerous studies have investigated the relationship between diet, dietary fiber, and diverticular disease (Aldoori et al. 1998; Crowe et al. 2011; Peery et al. 2013; Aune et al. 2020). However, the conclusions drawn from these studies remain controversial. For example, one study found a positive association between red meat intake and diverticulitis (Cao et al. 2018), while another study found no association between red meat intake and diverticulosis (Peery et al. 2012). Therefore, to investigate the impact of 20 diet‐related variables on intestinal diverticular disease (IDD), we conducted this Mendelian randomization (MR) analysis. MR, utilizing genetic variation as instrumental variables (IVs), is comparable to a randomized controlled trial in that the process of randomization of alleles at conception mirrors the randomization method used in a randomized controlled trial (Dictionary n.d.). Therefore, the utilization of MR analysis methods can generate robust and dependable conclusions by significantly mitigating the confounding variables and biases typically found in observational studies (Davey Smith and Hemani 2014).

## 2 Method

The MR study is based on the following premises: the independence assumption, where confounding variables have no effect on the instrumental variables (IVs); the relevance assumption, indicating a strong correlation between the exposure and the IVs; and the restriction assumption, asserting that the IVs affect the outcome solely through the exposure, without any other indirect pathways.

### 2.1 Data Sources

Twenty food categories were utilized as exposures in our investigation; these were mostly taken from the UK Biobank. These dietary categories include alcohol, meat, cheese, vegetables and fruits, bread and cereal, salt, coffee, water, and tea. Table 1 provides an overview of these exposures, while Table S1 details the specific measurement methods for each dietary category. The outcome of our investigation, IDD, was extracted directly from the FinnGen Biobank. It includes 33,618 European‐descent cases and 329,381 European‐descent controls. IDD was primarily defined based on hospital discharge data and cause of death records using ICD‐10 codes (K57) (WHO, 2024). Since the study data are publicly available and anonymized, an ethical review is unnecessary.

**TABLE 1:** Information on exposure and outcome datasets.

IEU GWAS id	Exposure or outcome	Participants included in analysis
ieu‐a‐835	Body mass index	322,154 European‐descent individuals
ieu‐b‐73	Alcoholic drinks per week	335,394 European‐descent individuals
ukb‐b‐5779	Alcohol intake frequency	462,346 European‐descent individuals
ukb‐b‐6324	Processed meat intake	461,981 European‐descent individuals
ukb‐b‐8006	Poultry intake	461,900 European‐descent individuals
ukb‐b‐2862	Beef intake	461,053 European‐descent individuals
ukb‐b‐17627	Nonoily fish intake	460,880 European‐descent individuals
ukb‐b‐2209	Oily fish intake	460,443 European‐descent individuals
ukb‐b‐5640	Pork intake	460,162 European‐descent individuals
ukb‐b‐14179	Lamb/mutton intake	460,006 European‐descent individuals
ukb‐b‐11348	Bread intake	452,236 European‐descent individuals
ukb‐b‐1489	Cheese intake	451,486 European‐descent individuals
ukb‐b‐8089	Cooked vegetable intake	448,651 European‐descent individuals
ukb‐b‐6066	Tea intake	447,485 European‐descent individuals
ukb‐b‐3881	Fresh fruit intake	446,462 European‐descent individuals
ukb‐b‐15926	Cereal intake	441,640 European‐descent individuals
ukb‐b‐1996	Salad/raw vegetable intake	435,435 European‐descent individuals
ukb‐b‐5237	Coffee intake	428,860 European‐descent individuals
ukb‐b‐16576	Dried fruit intake	421,764 European‐descent individuals
ukb‐b‐8121	Salt added to food	462,630 European‐descent individuals
ukb‐b‐14898	Water intake	427,588 European‐descent individuals
N/A	Diverticular disease of intestine	33,618 European‐descent cases and 329,381 European‐descent controls

*Note:* We present the IDs and sample sizes of 20 diet‐related factors from the IEU OpenGWAS project. More information about exposure and outcomes is available at the IEU OpenGWAS project (https://gwas.mrcieu.ac.uk/) and the FinnGen Biobank (https://r10.risteys.finngen.fi/).

Abbreviations: GWAS, genome‐wide association studies; IEU, Integrative Epidemiology Unit; NA, not applicable.

### 2.2 Selection of IVs

In Mendelian randomization, single‐nucleotide polymorphisms (SNPs) are utilized as IVs to infer potential causal relationships. For our study, SNPs were selected based on criteria including a 10,000 kb clumping window, significant linkage disequilibrium (*r* ^2^ < 0.001), and significance below the genome‐wide threshold (*p* < 5 × 10^−8^). To minimize bias and adhere to MR assumptions, SNPs showing significant association with the outcome (*p* < 5 × 10^−8^) were excluded from each analysis.

### 2.3 Statistical Analysis

Our study employed three widely recognized MR analysis techniques to assess causal relationships: the inverse‐variance‐weighted (IVW) method, chosen as the primary technique for its superior effectiveness in identifying causal relationships compared to the MR‐Egger and weighted median (WM) methods. The IVW method was chosen as the primary approach because of its higher statistical power and efficacy in detecting causal relationships, particularly when the assumption of no pleiotropy holds. By aggregating the weighted contributions from all SNPs, this method offers the most accurate causal estimate, provided the instrumental variables are valid. MR‐Egger, while less efficient than IVW, offers an advantage by accounting for pleiotropy, providing a consistent estimate even when some of the instruments are invalid. The weighted median method, on the other hand, allows for robustness by using the median of valid instrumental variables, offering a reliable causal estimate when up to 50% of the instruments are invalid. The IVW method must meet certain conditions to ensure the reliability of results: the requirement for horizontal pleiotropy to remain balanced or negligible (Hartwig, Davey Smith, and Bowden 2017). We have also conducted a multivariable Mendelian randomization analysis to reduce the potential impact of BMI. The false discovery rate (FDR) during multiple hypothesis testing can be reduced by applying FDR adjustments (Glickman, Rao, and Schultz 2014) to the *p*‐values obtained via the IVW method. This adjustment is particularly important in the context of MR studies, where a large number of genetic instruments are often tested simultaneously, increasing the likelihood of spurious associations due to the sheer volume of comparisons. We applied the MR‐PRESSO technique to detect and exclude outliers and then repeated all analyses. We measured heterogeneity utilizing Cochran's *Q*‐test. When heterogeneity was absent (*p* > 0.05), the fixed‐effects IVW method was adopted; otherwise, the random‐effects IVW method was adopted. The TwoSampleMR package (version 0.5.10) and MendelianRandomization package (version 0.9. 0) in R (version 4.3.2) were utilized in all statistical analyses.

## 3 Result

This study used Mendelian randomization to evaluate the effects of 20 diet‐related factors on IDD. Each component involved over 330,000 participants, providing a comprehensive analysis of the influence of dietary habits on IDD. As our previous research (Yang et al. 2023) demonstrated, F‐statistics exceeding 10 for each dietary component indicate a strong association between the IVs and these exposures. Weak instrument bias is regarded as being comparatively low when the F‐statistic is more than 10. Our study primarily focuses on the analytical outcomes of the IVW method, which is the most effective among the three methods for identifying causal relationships. After excluding SNPs associated with IDD, we analyzed the effects of 20 dietary factors on IDD and presented the results of the MR‐PRESSO method and three MR analysis methods in Table S2. Subsequently, we excluded outliers detected by the MR‐PRESSO method and presented the results after outlier exclusion in Table 2.

**TABLE 2:** The results of the three Mendelian randomization methods on the influence of dietary factors on diverticular disease of intestine after excluding outliers.

Outcome	Exposure	Number of SNPs	IVW_Beta	IVW_se	IVW_*p*val	IVW_*q*_value	Weighted_median_Beta	Weighted_median_se	Weighted_median_*p*val	MR_Egger_Beta	MR_Egger_se	MR_Egger_*p*val	egger_intercept	se	*p*
Diverticular disease of intestine	Alcoholic drinks per week	26	8.13E‐02	1.22E‐01	5.06E‐01	5.96E‐01	9.02E‐02	1.62E‐01	5.79E‐01	‐5.65E‐01	2.52E‐01	3.41E‐02	1.31E‐02	4.52E‐03	7.98E‐03
Diverticular disease of intestine	Alcohol intake frequency	85	6.53E‐02	5.89E‐02	2.68E‐01	4.87E‐01	7.34E‐02	7.92E‐02	3.54E‐01	4.48E‐02	1.85E‐01	8.09E‐01	5.06E‐04	4.33E‐03	9.07E‐01
Diverticular disease of intestine	Processed meat intake	21	−2.64E‐01	2.19E‐01	2.29E‐01	4.58E‐01	−4.38E‐01	2.33E‐01	6.02E‐02	1.05E‐02	1.10E+00	9.93E‐01	−4.18E‐03	1.64E‐02	8.02E‐01
Diverticular disease of intestine	Poultry intake	7	7.32E‐01	4.64E‐01	1.15E‐01	3.29E‐01	7.34E‐01	4.80E‐01	1.26E‐01	−1.27E+01	1.41E+01	4.07E‐01	1.46E‐01	1.52E‐01	3.82E‐01
Diverticular disease of intestine	Beef intake	10	9.62E‐02	3.62E‐01	7.90E‐01	7.90E‐01	−9.13E‐02	3.95E‐01	8.17E‐01	1.69E+00	2.08E+00	4.41E‐01	−1.95E‐02	2.51E‐02	4.59E‐01
Diverticular disease of intestine	Nonoily fish intake	10	7.09E‐01	3.53E‐01	4.49E‐02	2.12E‐01	5.12E‐01	3.94E‐01	1.94E‐01	3.06E+00	1.80E+00	1.28E‐01	−2.77E‐02	2.09E‐02	2.21E‐01
Diverticular disease of intestine	Oily fish intake	49	2.46E‐01	1.08E‐01	2.22E‐02	1.48E‐01	3.04E‐01	1.39E‐01	2.83E‐02	4.34E‐01	4.59E‐01	3.48E‐01	−2.76E‐03	6.54E‐03	6.74E‐01
Diverticular disease of intestine	Pork intake	9	1.06E+00	3.49E‐01	2.44E‐03	2.44E‐02	6.26E‐01	4.54E‐01	1.68E‐01	−1.47E+00	1.97E+00	4.80E‐01	2.51E‐02	1.93E‐02	2.34E‐01
Diverticular disease of intestine	Lamb/mutton intake	24	3.57E‐01	2.07E‐01	8.44E‐02	2.81E‐01	3.64E‐01	2.52E‐01	1.49E‐01	−8.90E‐01	9.80E‐01	3.74E‐01	1.36E‐02	1.05E‐02	2.07E‐01
Diverticular disease of intestine	Bread intake	24	1.69E‐01	2.10E‐01	4.20E‐01	5.96E‐01	−2.21E‐02	2.15E‐01	9.18E‐01	−7.31E‐01	9.60E‐01	4.54E‐01	1.31E‐02	1.36E‐02	3.47E‐01
Diverticular disease of intestine	Cheese intake	53	−3.98E‐02	1.20E‐01	7.40E‐01	7.90E‐01	1.05E‐01	1.36E‐01	4.37E‐01	−7.76E‐02	5.18E‐01	8.82E‐01	6.44E‐04	8.59E‐03	9.41E‐01
Diverticular disease of intestine	Cooked vegetable intake	12	−1.13E‐01	3.73E‐01	7.62E‐01	7.90E‐01	−3.82E‐01	3.95E‐01	3.34E‐01	−7.66E‐01	4.01E+00	8.52E‐01	6.74E‐03	4.11E‐02	8.73E‐01
Diverticular disease of intestine	Tea intake	32	−1.04E‐01	1.22E‐01	3.93E‐01	5.96E‐01	2.38E‐02	1.34E‐01	8.59E‐01	−9.60E‐02	2.72E‐01	7.27E‐01	−1.78E‐04	5.29E‐03	9.73E‐01
Diverticular disease of intestine	Fresh fruit intake	48	−3.84E‐01	1.98E‐01	5.30E‐02	2.12E‐01	−3.43E‐01	2.43E‐01	1.59E‐01	2.62E‐01	6.68E‐01	6.97E‐01	−6.22E‐03	6.14E‐03	3.17E‐01
Diverticular disease of intestine	Cereal intake	29	1.07E‐01	1.59E‐01	4.98E‐01	5.96E‐01	1.34E‐02	2.00E‐01	9.47E‐01	2.13E‐02	6.52E‐01	9.74E‐01	1.26E‐03	9.21E‐03	8.92E‐01
Diverticular disease of intestine	Salad/raw vegetable intake	10	−5.07E‐01	3.93E‐01	1.97E‐01	4.58E‐01	−4.33E‐01	4.47E‐01	3.33E‐01	−1.39E+00	1.95E+00	4.96E‐01	9.54E‐03	2.06E‐02	6.56E‐01
Diverticular disease of intestine	Coffee intake	34	−1.24E‐01	1.64E‐01	4.50E‐01	5.96E‐01	3.08E‐02	1.52E‐01	8.40E‐01	1.22E‐01	3.27E‐01	7.12E‐01	−4.74E‐03	5.44E‐03	3.90E‐01
Diverticular disease of intestine	Dried fruit intake	33	−9.53E‐01	1.80E‐01	1.16E‐07	2.31E‐06	−9.56E‐01	2.19E‐01	1.22E‐05	−1.72E+00	8.38E‐01	4.89E‐02	9.37E‐03	1.00E‐02	3.57E‐01
Diverticular disease of intestine	Salt added to food	83	1.08E‐01	8.73E‐02	2.15E‐01	4.58E‐01	1.08E‐02	1.19E‐01	9.28E‐01	−7.69E‐02	2.82E‐01	7.86E‐01	2.77E‐03	4.00E‐03	4.91E‐01
Diverticular disease of intestine	Water intake	33	1.33E‐01	1.90E‐01	4.84E‐01	5.96E‐01	−4.24E‐02	1.77E‐01	8.11E‐01	−1.36E‐01	5.32E‐01	8.00E‐01	4.26E‐03	7.88E‐03	5.92E‐01

*Note:* We analyzed the relationship between 20 dietary factors and diverticular disease of intestine. The IVW method, known for its heightened sensitivity in detecting causality, serves as our primary tool to ascertain the presence of a causal relationship. The MR‐Egger method is also employed for detecting horizontal pleiotropy due to its allowance for the presence of nonzero intercepts (the columns in the table corresponding to “egger_intercept,” “se,” and “*p*val”), and a *p*val below 0.05 suggests the existence of horizontal pleiotropy. *q* value: The *p* value post‐FDR method (Benjamini and Hochberg) corrected.

Abbreviations: IVW, inverse‐variance‐weighted method; MR_Egger, MR‐Egger method; *p*val, *p*‐value; SNPs, single‐nucleotide polymorphisms; Weighted_median, weighted median method.

In our analysis, we detected horizontal pleiotropy when analyzing the effects of weekly alcohol consumption on IDD after excluding outliers (which were not detected before exclusion). Therefore, we considered this analysis invalid. According to the IVW method, among the remaining 19 diet‐related factors, we found that the intake of nonoily fish (*β* = 0.709, *p* = 0.0449), the intake of oily fish (*β* = 0.246, *p* = 0.0222), and the intake of pork (*β* = 1.06, *p* = 0.00244) are risk factors for IDD, while the intake of dried fruit (*β* = −0.953, *p* < 0.0001) was identified as a protective factor against IDD. Of these four dietary factors, only the intake of pork (*q* value = 0.0244) and the intake of dried fruit (*q* value < 0.0001) remained significant after FDR correction. As shown in Table 3, we performed a multivariable Mendelian randomization analysis of these four diet factors associated with IDD, adjusting for BMI. We found that, after considering the impact of BMI, only the intake of dried fruit (*β* = −0.764, *p* = 0.00343) remained significantly associated with IDD. The other 15 diet‐related factors—alcohol intake frequency (*β* = 0.0653, *p* = 0.268), the intake of processed meat (*β* = −0.264, *p* = 0.229), poultry intake (*β* = 0.732, *p* = 0.115), beef intake (*β* = 0.0962, *p* = 0.790), lamb/mutton intake (*β* = 0.357, *p* = 0.0844), bread intake (*β* = 0.169, *p* = 0.420), cheese intake (*β* = −0.0398, *p* = 0.740), cooked vegetable intake (*β* = −0.113, *p* = 0.762), tea intake (*β* = −0.104, *p* = 0.393), fresh fruit intake (*β* = −0.384, *p* = 0.0530), cereal intake (*β* = 0.107, *p* = 0.498), raw vegetable intake (*β* = −0.507, *p* = 0.197), coffee intake (*β* = −0.124, *p* = 0.450), salt added to food (*β* = 0.108, *p* = 0.215), and water intake (*β* = 0.133, *p* = 0.484)—showed no association with IDD. According to the WM method, among these 19 diet‐related factors, only oily fish intake (*β* = 0.304, *p* = 0.0283) and dried fruit intake (*β* = −0.956, *p* < 0.0001) were associated with IDD. According to the MR‐Egger method, only dried fruit intake (*β* = −1.72, *p* = 0.0489) among these 19 diet‐related factors was associated with IDD. Further information on these MR analysis methods can be found in Table 2.

**TABLE 3:** The multivariable Mendelian randomization analysis of the four dietary factors associated with diverticular disease accounted for the potential confounding effect of BMI.

Outcome	Exposure	Beta	Se	*p*
Diverticular disease of intestine || id:K11_DIVERTIC	Body mass index || id:ieu‐a‐835	1.57E‐01	6.70E‐02	1.88E‐02
	Nonoily fish intake || id:ukb‐b‐17627	1.25E‐01	3.91E‐01	7.49E‐01
Diverticular disease of intestine || id:K11_DIVERTIC	Body mass index || id:ieu‐a‐835	1.49E‐01	6.54E‐02	2.28E‐02
	Oily fish intake || id:ukb‐b‐2209	4.92E‐03	1.99E‐01	9.80E‐01
Diverticular disease of intestine || id:K11_DIVERTIC	Body mass index || id:ieu‐a‐835	1.74E‐01	6.57E‐02	8.05E‐03
	Pork intake || id:ukb‐b‐5640	‐8.84E‐02	4.35E‐01	8.39E‐01
Diverticular disease of intestine || id:K11_DIVERTIC	Body mass index || id:ieu‐a‐835	1.45E‐01	7.67E‐02	5.92E‐02
	Dried fruit intake || id:ukb‐b‐16576	−7.64E‐01	2.61E‐01	3.43E‐03

*Note:* We conducted separate multivariable Mendelian randomization analyses for the four dietary factors and body mass index (BMI) to eliminate the potential influence of BMI on the relationship between diet and diverticular disease.

## 4 Discussion

This research, assessing the influence of 20 diet‐related factors on IDD, indicates varied impacts of different diets on the disease. Through our analysis, we found that fish intake and pork intake increase the risk of IDD, while dried fruit intake decreases the risk of IDD. In our study, we detected horizontal pleiotropy when analyzing the relationship between weekly alcohol intake and IDD. The presence of horizontal pleiotropy violates the restriction assumption of MR (potentially influencing the outcome through other pathways) (Dictionary n.d.), and we therefore consider this analysis invalid. Nevertheless, when horizontal pleiotropy is not considered, weekly alcohol intake does not show a correlation with IDD. Our study also analyzed the impact of alcohol intake frequency on IDD and found no association. Therefore, we conclude that alcohol consumption and IDD are not related. Currently, numerous studies have investigated the relationship between alcohol consumption and diverticular disease; however, their connection has not been fully elucidated. Some studies have found that alcohol consumption is a risk factor for developing diverticulosis (Sharara et al. 2013; Yan, Wu, and Pan 2022). A study, however, has found no association between alcohol consumption and symptomatic diverticular disease (Aldoori et al. 1995). A meta‐analysis also did not observe an increased risk of diverticulosis among study participants (Jaruvongvanich, Sanguankeo, and Upala 2017). We found no correlation between alcohol consumption and diverticular disease. Mendelian randomization (MR) effectively mitigates the influence of confounding factors and reverse causation. Importantly, employing the MR analysis method allowed us to include over 30,000 cases of diverticular disease in our study. Although the impact of IDD is relatively smaller compared to infectious diseases and other conditions (Meng et al. 2024, 2023, 2024), understanding the effect of diet on IDD remains of significant importance.

Some studies currently find an association between red meat consumption and diverticulitis (Cao et al. 2018; Aldoori et al. 1994). According to a U.S. study involving 46,461 males, red meat consumption is associated with an increased incidence of diverticulitis (Cao et al. 2018). Red meat consumption was reported by Aldoori and colleagues to have a strong correlation with an elevated risk of diverticular disease (Aldoori et al. 1994). However, another study has found conflicting conclusions: Peery et al. (2012) observed no correlation between the amount of red meat consumed and diverticulosis detected during colonoscopy. In our study, we found that among the three types of red meat (pork, lamb, and beef), only pork consumption was associated with an increased risk of IDD. Fish consumption, regardless of whether it was oily or nonoily fish, was also associated with an increased risk of IDD. However, the intake of processed meats and poultry was not associated with IDD risk.

A study from Sweden found that cereal intake was not associated with the risk of hospitalization for diverticular disease (Mahmood et al. 2019). Our study also showed that cereal intake was not associated with the risk of IDD. Multiple studies have explored the relationship between the intake of vegetables and fruits and the risk of diverticular disease (Aune et al. 2020; Mahmood et al. 2019; Maxner et al. 2020; Aldoori et al. 1994; Crowe et al. 2014). A rich fruit and vegetable diet may lower the risk of diverticular disease‐related hospitalization (Mahmood et al. 2019). Based on colonoscopy findings, a study found that both cereal and vegetable intake were negatively correlated with the risk of developing diverticular disease (Maxner et al. 2020). A meta‐analysis found that for every 10 g/day increase in dietary fiber from fruits, the risk of diverticular disease decreased by 44%, whereas dietary fiber from vegetables showed no association with the risk of diverticular disease (Aune et al. 2020). A study from the United States also found a negative correlation between total dietary fiber intake and the risk of diverticular disease, primarily attributed to the effects of fruits and vegetables (Aldoori et al. 1994). A large prospective study from the United Kingdom found a negative correlation between dietary fiber intake from fruits and the risk of diverticular disease, while fiber intake from vegetables showed no association with diverticular disease (Crowe et al. 2014). Our study found that vegetable intake, whether raw or cooked, was not associated with IDD. However, dried fruit intake significantly reduced the risk of IDD. Fresh fruit intake was not associated with IDD, though it showed a trend toward reducing the risk of IDD (*β* = −0.384, *p* = 0.0530). Multiple studies have indicated that coffee (caffeine) consumption does not increase the risk of developing diverticular disease (Aldoori et al. 1995; Yuan and Larsson 2022), which is consistent with our findings. Our study employed MR to analyze the relationship between 20 diet‐related factors and IDD. Through our analysis, we systematically evaluated the association between diet and IDD.

It is undeniable that this research has inherent limitations. Firstly, our capacity for conducting additional research is constrained by the inability to further segment the dietary components examined in our study. This limitation hampers our ability to explore the nuanced relationships and subtle differences between various dietary components. Secondly, the absence of summary‐level GWAS data categorized by age and gender precludes conducting stratified analyses based on these variables. This limitation inhibits our ability to investigate genetic associations across different age groups and genders. Thirdly, the generalizability of our findings to other populations is uncertain due to the predominance of exposure and outcome data collected from European populations.

## 5 Conclusion

Our analysis provides compelling evidence that specific dietary factors influence the risk of intestinal diverticular disease (IDD). Notably, pork and fish intake were identified as risk factors, whereas dried fruit intake was found to be protective against IDD. No significant associations were observed for the other dietary components examined. These findings underscore the importance of dietary modifications in the prevention and management of IDD and highlight the potential for further research to explore these associations in diverse populations.

## Author Contributions

**Wangzhao Tian:** conceptualization (equal), data curation (equal), investigation (equal), resources (equal), software (equal), supervision (equal), validation (equal), visualization (equal), writing – review and editing (equal). **Wenwen Yang:** conceptualization (equal), data curation (equal), formal analysis (equal), funding acquisition (equal), investigation (equal), methodology (equal), project administration (equal), resources (equal), software (equal), supervision (equal), validation (equal), visualization (equal), writing – original draft (equal), writing – review and editing (equal). **Xiaorui Wang:** conceptualization (equal), data curation (equal), formal analysis (equal), funding acquisition (equal), investigation (equal), methodology (equal), project administration (equal), resources (equal), software (equal), supervision (equal), validation (equal), visualization (equal), writing – original draft (equal). **Yanjiang Yang:** conceptualization (equal), data curation (equal), formal analysis (equal), funding acquisition (equal), investigation (equal), methodology (equal), project administration (equal), resources (equal), software (equal), supervision (equal), validation (equal), visualization (equal), writing – original draft (equal), writing – review and editing (equal).

## Conflicts of Interest

The authors declare no conflicts of interest.

## Supporting information


Data S1.

## Data Availability

All GWAS data used in this study are available in the IEU open GWAS project (https://gwas.mrcieu.ac.uk/) and the FinnGen Biobank (https://r10.risteys.finngen.fi/).
